# HGF Modulates Actin Cytoskeleton Remodeling and Contraction in Testicular Myoid Cells

**DOI:** 10.3390/biomedicines3010089

**Published:** 2015-01-28

**Authors:** Angela Catizone, Giulia Ricci, Maria Caruso, Michela Galdieri, Katia Corano Scheri, Virginia Di Paolo, Rita Canipari

**Affiliations:** 1 Department of Anatomy, Histology, Forensic Medicine and Orthopedics, Section of Histology and Embryology, Sapienza University of Rome, Via A. Scarpa 16, 00161 Rome, Italy; E-Mails: angela.catizone@uniroma1.it (A.C.); maria.caruso@uniroma1.it (M.C.); katia.corano@gmail.com (K.C.S.); virginia.dipaolo@uniroma1.it (V.D.P.); rita.canipari@uniroma1.it (R.C.); 2 Department of Experimental Medicine, Histology and Embryology Laboratory, School of Medicine, Second University of Naples, Via Luciano Armanni 5, 80138 Naples, Italy; E-Mail: michela.galdieri@uniroma1.it

**Keywords:** myoid cells, HGF/c-Met, uPA, actin cytoskeleton, TGF-β

## Abstract

The presence of the HGF/Met system in the testicular myoid cells was first discovered by our group. However, the physiological role of this pathway remains poorly understood. We previously reported that HGF increases uPA secretion and TGF-β activation in cultured tubular fragments and that HGF is maximally expressed at Stages VII–VIII of the seminiferous epithelium cycle, when myoid cell contraction occurs. It is well known that the HGF/Met pathway is involved in cytoskeletal remodeling; moreover, the interaction of uPA with its receptor, uPAR, as well as the activation of TGF-β have been reported to be related to the actin cytoskeleton contractility of smooth muscle cells. Herein, we report that HGF induces actin cytoskeleton remodeling *in vitro* in isolated myoid cells and myoid cell contraction in cultured seminiferous tubules. To better understand these phenomena, we evaluated: (1) the regulation of the uPA machinery in isolated myoid cells after HGF administration; and (2) the effect of uPA or Met inhibition on HGF-treated tubular fragments. Because uPA activates latent TGF-β, the secretion of this factor was also evaluated. We found that both uPA and TGF-β activation increase after HGF administration. In testicular tubular fragments, HGF-induced TGF-β activation and myoid cell contraction are abrogated by uPA or Met inhibitor administration.

## 1. Introduction

Hepatocyte growth factor (HGF) is a pleiotropic cytokine originally purified as a potent mitogen for hepatocytes [1,2] and subsequently identified as a “scatter factor” [3,4] for its ability to disperse sheets of contiguous epithelial cells [3]. HGF is secreted as an inactive, single-chain precursor that is cleaved to acquire its active form. HGF activation can be provided by the HGF activator protein (HGFA), a serine protease able to cleave immature HGF precursor to form the mature bioactive protein, or by active metalloproteinases (MMP2 and MMP9), as well as by plasminogen activator (PA) [5].

The action of HGF is mediated by a specific receptor, Met. This receptor is normally expressed by epithelial cells, whereas HGF production has been mainly found to be restricted to cells of mesenchymal origin. These findings suggest a relevant role for HGF in mesenchymal-epithelial interactions [6]. We previously reported that HGF and Met are expressed in the postnatal rat testis and influence many functional activities of testicular somatic and germ cells (for a review, see [7]). In myoid cells, HGF is expressed at both prenatal and postnatal ages, whereas Met is expressed by myoid cells only during postnatal rat testis development, indicating that this lineage becomes sensitive to HGF only postnatally [8,9,10]. In Sertoli cells, Met is absent up to 20–25 days postpartum, but low levels of expression begin at puberty [10]. Interestingly, in adult rats, HGF is maximally expressed at Stages VII–VIII of the seminiferous epithelium cycle, when germ cells traverse the blood–testis barrier (BTB) and spermiation occurs, whereas its level falls in the subsequent Stages IX–XII and XIII–I [11].

Myoid cells represent an interesting model of smooth muscle cells that contain desmin-type intermediate filaments [12] and α-smooth muscle isoactin as a specific differentiation marker [13]. Myoid cells surrounding the seminiferous tubule are responsible for tubular contractility, which is important for the progression of spermatozoa toward the rete testis. The regulation of myoid cell contractility occurs under endocrine-paracrine control. A variety of substances have been shown to affect myoid cell activity, including oxytocin [14], vasopressin [15], prostaglandins [16] and endothelin [17,18].

We previously reported that HGF is involved in the control of peritubular myoid cell spreading *in vitro* [8]. Interestingly, in the seminiferous tubule compartment, we have shown that HGF is mainly produced by myoid cells and adult Sertoli cells [8,19]. Moreover, we demonstrated that HGF increases the amount of the secreted TGF-β active fraction [20,21] in cultured seminiferous tubules. Intriguingly, it has been reported that TGF-β induces the contractility of smooth muscle cells [22] and that HGF might have an indirect effect on TGF-β by affecting molecules, such as plasminogen activator (PA), that are able to activate the latent form of the protein [23].

Plasminogen activators (PAs) are serine proteases that cleave the proenzyme, plasminogen, which is present in plasma and extracellular fluids, into the active protease plasmin. Two forms of PA, urokinase type (uPA) and tissue type (tPA), have been characterized in mammals; these proteins have different catalytic and antigenic properties and are encoded by two distinct genes [24]. These proteases are produced by a large number of cell and tissue types and are involved in physiological and pathological processes that require localized and controlled proteolysis [25] and/or local activation of growth factors [26]. Beyond the regulation of proteolysis, uPA is implicated in processes, such as cell migration, adhesion and proliferation and vascular remodeling [27]. uPA-induced signal transduction can be mediated via a specific receptor (uPAR), and a parallel between the remodeling of the actin cytoskeleton of smooth muscle cells and the interaction of uPA and its receptor, uPAR, has been reported in the literature [28,29].

To increase our knowledge on the effects exerted by HGF in the control of myoid cell functions, in the present paper, we investigated the capability of this growth factor to remodel the actin cytoskeleton of myoid cells and consequently modulate peritubular contractility.

To this aim, we analyzed the morphological effect of HGF treatment on isolated myoid cells and on seminiferous tubules isolated from the testes of animals at different postnatal ages. Moreover, we evaluated the gene expression of *tPA*, *uPA*, the uPA receptor, *uPAR*, and the uPA inhibitor, *PAI-1*, in control conditions and after HGF administration in isolated myoid cells. In the same culture conditions, the secretion of uPA, tPA and PAI-1 was also quantified. Finally, we observed the effect of uPA inhibition on the cytoskeleton of HGF-treated myoid cells in seminiferous tubule organ cultures. The effect of Met inhibition on myoid cell contraction and on TGF-β activation in cultured tubular fragments has been also evaluated.

## 2. Results

### 2.1. Effect of HGF on Cell Shape and the Actin Cytoskeleton of Cultured Myoid Cells

**Figure 1:**
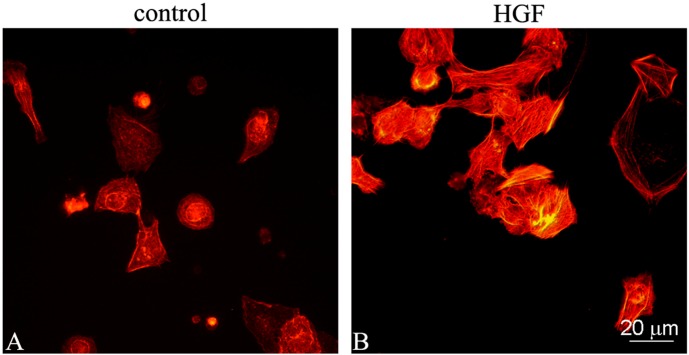
F-actin distribution pattern in cultured myoid cells purified from prepubertal rat testes. The figure shows the F-actin cytoskeletal pattern of purified myoid cells cultured for 24 h in medium alone (control) (**A**) or in the presence of 150 U/mL (45 ng/mL) HGF (**B**).

It has been previously demonstrated that the morphology of myoid cells cultured in the presence of HGF changes from a round shape to an elongated, enlarged shape [7]. To understand whether this change in cell morphology was associated with actin fiber polymerization and cytoskeletal rearrangement, myoid cells were stained with rhodamine-labeled phalloidin. In the control cells, the characteristic meshwork of actin filaments was observed (Figure 1A). The addition of 150 U/mL (45 ng/mL) HGF resulted in a rearrangement of the cytoskeleton, with the actin filaments assembled to form bundles of stress fibers (Figure 1B).

### 2.2. Effect of HGF on Myoid Cells Organized in Isolated Seminiferous Tubules

**Figure 2:**
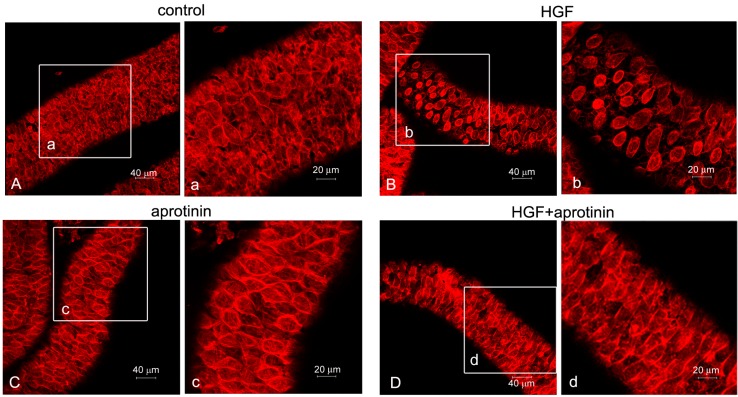
Confocal analysis of F-actin cytoskeletal modifications in cultured prepubertal rat seminiferous tubules. The figure shows the F-actin cytoskeletal pattern of prepubertal rat seminiferous tubules cultured for 24 h in medium alone (control) (**A**,**a**) or in the presence of 150 U/mL (45 ng/mL) HGF (**B**,**b**), 15 μM aprotinin (**C**,**c**) or HGF + aprotinin (**D**,**d**). Lower magnification (**A**–**D**) and higher magnification (**a**–**d**) of the images are shown.

**Figure 3:**
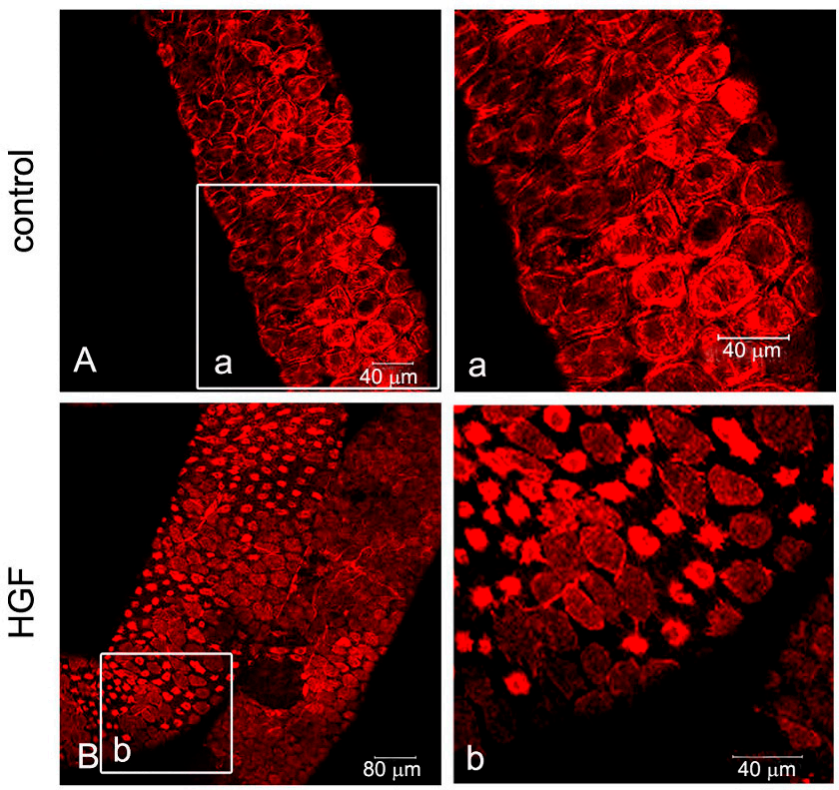
Confocal analysis of F-actin cytoskeletal modifications in cultured pubertal rat seminiferous tubules. The figure shows the F-actin cytoskeletal pattern of pubertal rat seminiferous tubules cultured for 24 h in medium alone (control) (**A**,**a**) or in the presence of HGF (**B**,**b**). Lower magnification (**A**,**B**) and higher magnification (**a**,**b**) of the images are shown.

**Figure 4:**
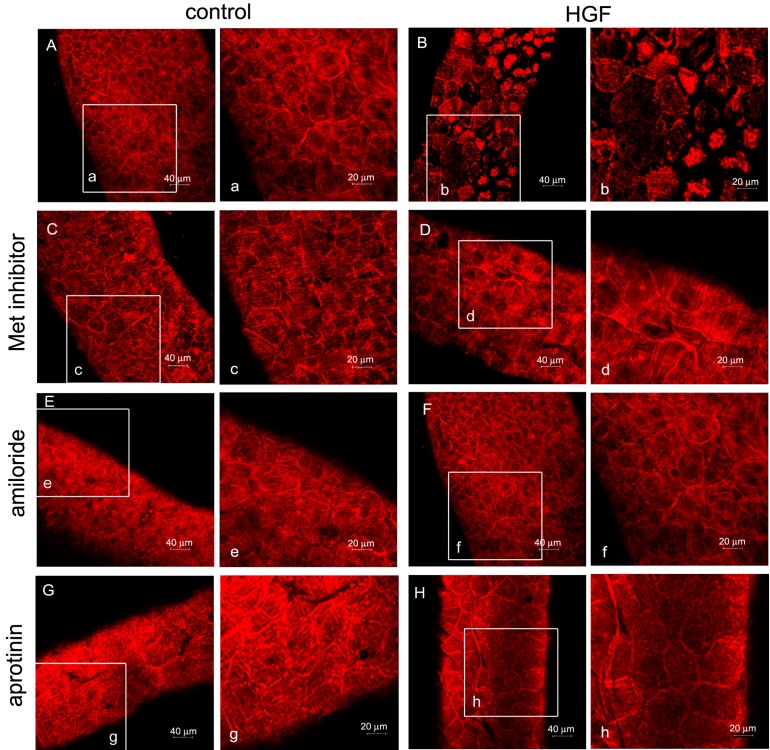
Confocal analysis of F-actin cytoskeletal modifications in cultured adult rat seminiferous tubules isolated at Stages VI–VIII. The figure shows the F-actin cytoskeletal pattern of adult rat seminiferous tubules (Stages VI–VIII) cultured for 24 h in medium alone (control) (**A**,**a**) or in the presence of HGF (**B**,**b**), Met-inhibitor (**C**,**c**), HGF + Met-inhibitor (**D**,**d**), amiloride (**E**,**e**), HGF + amiloride (**F**,**f**), aprotinin (**G**,**g**) and HGF + aprotinin (**H**,**h**). Lower magnification (**A**–**H**) and higher magnification (**a**–**h**) of the images are shown.

Seminiferous tubules were isolated from rats of different ages (17/18, 35 and 60 days) and cultured in the presence or absence of 150 U/mL (45 ng/mL) HGF. At the end of incubation, the actin cytoskeleton was stained with rhodamine-labeled phalloidin, and the seminiferous tubules were observed with a confocal microscope. In all of the different ages examined, the myoid cells were arranged in a continuous layer of epithelioid polygonal cells with the actin cytoskeleton showing a characteristic orthogonal meshwork (Figure 2A, Figure 3A and Figure 4A). The exposure of tubules to 150 U/mL (45 ng/mL) HGF resulted in a change in the cytoskeleton organization, with the centrally-located fibers forming evident rings. In prepubertal animals (Figure 2B), this pattern of response was observed in almost the whole peritubulum, whereas in pubertal rats, an evident spotted pattern was observed, although with a stronger response (Figure 3B(b)). To understand the mechanism behind this differential response, we performed the same experiments utilizing adult rats, where it is possible to distinguish the different stages of the seminiferous epithelium cycle and where myoid cell contraction starts to be physiologically important. In HGF-treated tubules, we observed the spotted pattern of cytoskeletal organization, which was similar to the tubules isolated from pubertal animals, exclusively at Stages VI-VIII. A representative confocal image of this stage is shown in Figure 4. In the other stages no differences in the morphology of peritubular myoid cells were observed after HGF treatment. To test the specificity of the reported HGF -dependent myoid cell contraction, we performed the same experiments on adult testis tubular fragments using the Met-specific inhibitor, PF-04217903. As expected, the Met inhibitor administration abrogated the HGF-dependent myoid cell contraction (Figure 4C,D).

### 2.3. Presence of MET

**Figure 5:**
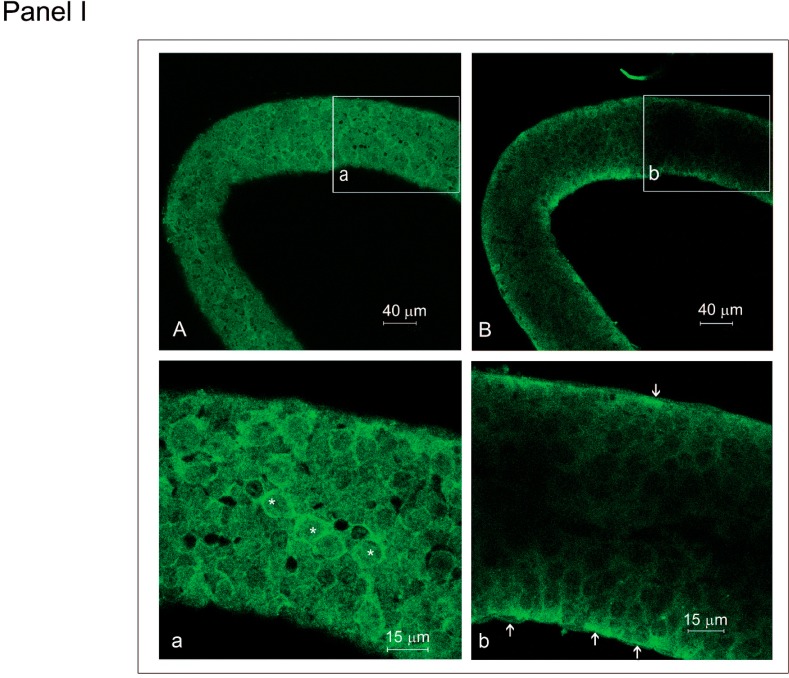

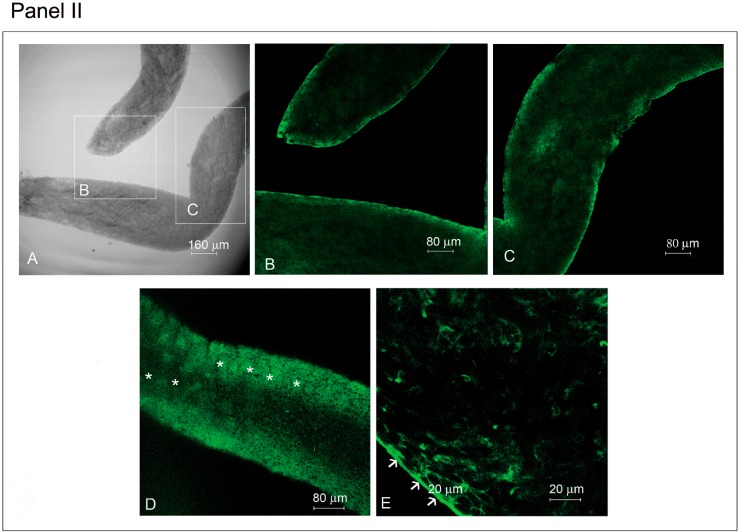
Confocal analysis of Met immunolocalization in prepubertal and adult rat seminiferous tubules. (**Panel I**): (**A**) Met distribution pattern in the peritubular compartment is shown. (**a**) Higher magnification of the square drawn in (**A**); asterisks indicate myoid cells. (**B**) Met distribution pattern imaged at the internal level of the same seminiferous tubule of (**A**). (**b**) Higher magnification of the square drawn in (**B**); arrows indicate myoid cells; (**Panel II**): (**A**) Transmission bright field images of seminiferous tubules at Stages VI–VIII (**B**) and I–V (**C**). (**B**,**C**) Relative Met distribution pattern at the internal level of the tubules shown in the squares in (**A**). (**D**) Met distribution pattern imaged at the peritubular myoid level of a seminiferous tubule at Stages VI–VIII. Asterisks indicate myoid cells. (**E**) Met distribution pattern imaged at the internal level of a seminiferous tubule at Stage X. Arrows indicate myoid cells.

The expression of *MET* mRNA in the myoid cells during postnatal testis development has been previously demonstrated by northern blot analysis [8,10]. However, to test whether the differential response to HGF at the different ages and at different epithelium seminiferous stages was due to a non-homogeneous distribution of the HGF receptor, Met, tubules isolated from the testes of prepubertal (Figure 5, Panel I) or adult (Figure 5, Panel II) rats were analyzed by immunofluorescence with anti-Met antibody. Confocal microscopy analysis showed that the myoid cell layer of the seminiferous tubules was always positive for Met, and the staining was independent of the age and the stage of the cycle (Figure 5). However, the intensity of Met positivity among neighboring myoid cells was variable, although it was not possible to relate this parameter to the age or the stage of the cycle.

### 2.4. Effect of Inhibition of PA Activity on Actin Cytoskeleton Remodeling

Urokinase PA and the interaction with its receptor, uPAR, have been implicated in actin cytoskeleton organization and adhesion capacity in vascular smooth muscle cells [29]. To test whether the observed effects of HGF on myoid cell shape and actin cytoskeleton rearrangement were mediated by PA activity, tubules isolated from prepubertal and adult rats were cultured for 24 h in the presence of 15 μM aprotinin alone or in combination with 150 U/mL (45 ng/mL) HGF. As shown in Figure 2C and Figure 4G, aprotinin alone did not affect the actin cytoskeleton, but the combination of aprotinin and HGF almost completely abolished the effect of HGF (Figure 2D and Figure 4H). To test the specificity of the results obtained, we performed the same experiments on tubules isolated from adult rats with another PA inhibitor (amiloride), obtaining overlapping results (Figure 4E,F).

### 2.5. Effects of HGF on PA Activity in Cultured Rat Myoid Cells

**Figure 6:**
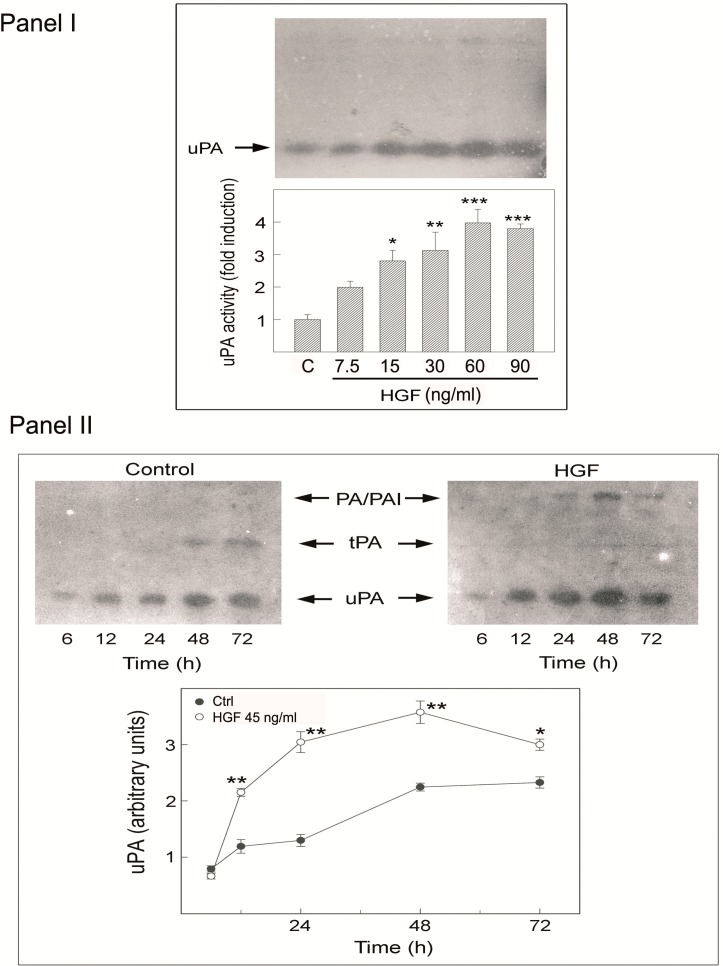

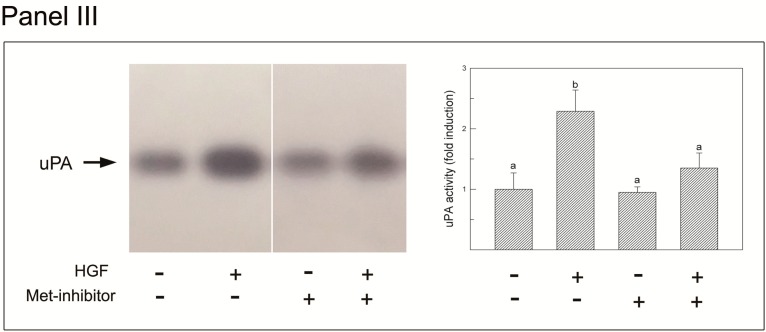
HGF mediated a uPA secretion increase in purified myoid cells and in cultured seminiferous tubules. (**Panel I**) Dose-response curve of uPA secreted by purified myoid cells cultured for 24 h in the presence of increasing doses of HGF (25–300 U/mL; 7.5–90 ng/mL). Aliquots of conditioned medium were analyzed by SDS-PAGE followed by zymography. The bands were analyzed by densitometry. Values represent the mean ± SEM of three separate experiments and are expressed as fold induction relative to control, which was set equal to one. * *p* < 0.05; ** *p* < 0.01 and *** *p* < 0.001; (**Panel II**) Time course of PA production by purified myoid cells cultured in medium alone (**left**) or in the presence of 150 U/mL (45 ng/mL) HGF (**right**). The uPA bands were analyzed by densitometry. Values represent the mean ± SEM of three separate experiments and are expressed as arbitrary units. * *p* < 0.05; ** *p* < 0.01; (**Panel III**) uPA secreted by tubular fragments cultured for 24 h in control condition, with Met inhibitor alone or in combination with HGF. Values are expressed as arbitrary units. a *vs.* b *p* < 0.05. The same letters indicate values that are not statistically significant.

To evaluate the effect of HGF on myoid cell PA secretion, the cells were incubated for 24 h in MEM supplemented with 0.1% BSA in the presence of different concentrations of HGF (25–300 U/mL; 7.5–90 ng/mL). The culture medium was then analyzed for PA activity by zymography. The results of a representative experiment are shown in Figure 6 (Panel I). A band with an apparent molecular mass of approximately 45 kDa, corresponding to rat uPA, was the predominant enzyme present in the conditioned medium (Figure 6, Panel I). After HGF stimulation, uPA secretion was increased in a dose-dependent manner. 

PA activity was quantified by densitometric scanning of zymographies, and the values are expressed as fold induction with respect to the values of the controls, which were set equal to one (Figure 6, Panel I). For the subsequent experiments, a dose of 150 U/mL (45 ng/mL) HGF was used.

The effect of HGF on PA secretion was also evaluated at different culture times. Myoid cells were cultured in medium alone (control) or with 150 U/mL (45 ng/mL) HGF for the times indicated in Panel II of Figure 6. In HGF-treated cultures, there was a significant increase in uPA production, which reached a plateau at 48 h, whereas in the control conditions, the increase was less evident. At these longer times of culture, a band with an apparent molecular mass of approximately 70 kDa corresponding to rat tPA became evident in the control conditions. However, after hormonal stimulation, we observed a decrease in tPA production. In all zymographies of HGF-treated cells, a band with a higher molecular weight appeared, suggesting the presence of a PA–PAI complex. Urokinase PA activity was quantified by densitometric scanning of zymographies, and the values are expressed as arbitrary units (Figure 6, Panel II).

### 2.6. Effects of Met Inhibition on PA Activity in Cultured Adult Seminiferous Tubule Fragments

We previously demonstrated that HGF administration to tubules at Stages VII–VIII is able to increase the uPA level [11]. Herein, we report that the selective inhibition of Met by PF-04217903 is able to abrogate this effect, indicating the specific and direct role of HGF on uPA modulation (Figure 6, Panel III).

### 2.7. Hormonal Regulation of tPA, uPA, PAI-1 and uPAR mRNA Levels

**Figure 7:**
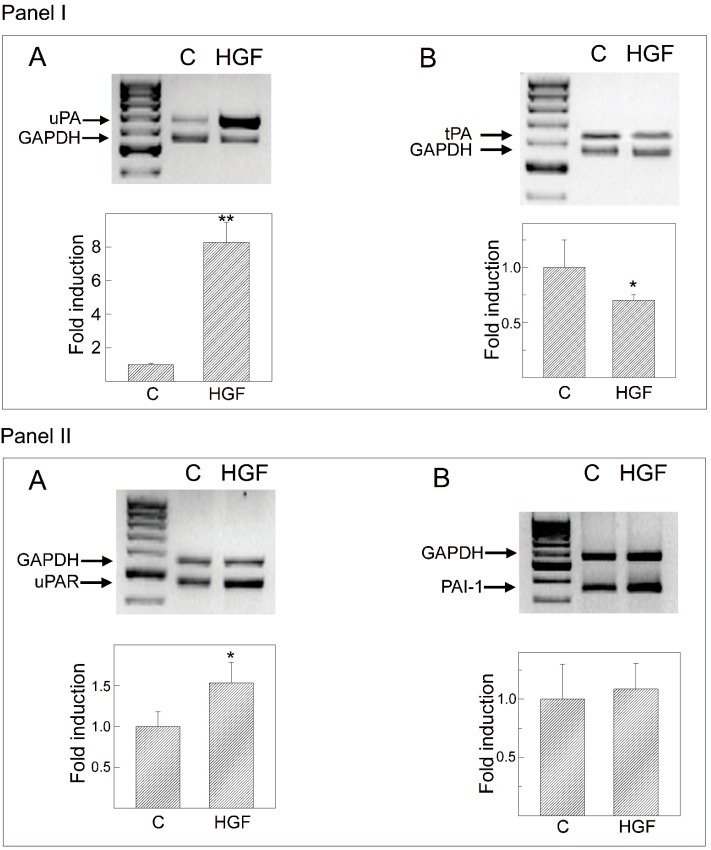
*uPA*, *tPA*, *uPAR* and *PAI-1* gene expression level after HGF administration. (**Panel I**): The effect of HGF stimulation on *uPA* (**A**) and *tPA* (**B**) gene expression, as detected by multiplex RT-PCR. Myoid cells were cultured for 24 h in medium alone C or in the presence of 150 U/mL (45 ng/mL) HGF. Total RNA was subjected to RT-PCR, and aliquots of the PCR products were electrophoresed on a 1.5% agarose gel (**top**). The values of the uPA and tPA PCR products were normalized to their corresponding GAPDH values and are expressed as fold induction with respect to C, which was arbitrarily set to one. Values represent the mean ± SEM of three separate experiments. * *p* < 0.05; ** *p* < 0.001. (**Panel II**): The effect of HGF stimulation on *uPAR* (**A**) and *PAI-1* (**B**) gene expression as detected by multiplex RT-PCR. Myoid cells were cultured for 24 h in medium alone C or in the presence of 150 U/mL (45 ng/mL) HGF. Total RNA was subjected to RT-PCR, and aliquots of the PCR products were electrophoresed on a 1.5% agarose gel (**top**). The values of uPAR and PAI-1 PCR products were normalized to their corresponding GAPDH values and are expressed as fold induction with respect to C, which was arbitrarily set to one. Values represent the mean ± SEM of three separate experiments. * *p* < 0.05.

To determine whether the effects of HGF on PA activity were associated with changes in mRNA levels, total RNA was extracted from myoid cells cultured for 24 h in the presence or absence of 150 U/mL (45 ng/mL) HGF and analyzed by multiplex PCR (Figure 7).

A densitometric analysis of the bands revealed that after hormonal stimulation, there was an 8.3-fold increase in *uPA* mRNA levels, whereas *tPA* mRNA decreased by 30% compared with the values found in basal conditions (Figure 7, Panel I). The observed levels of mRNA mirrored the enzymatic activity of both tPA and uPA in the culture medium (Figure 6, Panel II).

Urokinase PA activity is regulated by the binding of uPA to its receptor, uPAR, and by the inhibitor, PAI-1. Therefore, we also analyzed the levels of *uPAR* and *PAI-1* mRNA. As shown in Panel II of Figure 7, HGF induced a significant increase in *uPAR* mRNA (50%), but did not significantly change the levels of *PAI-1* mRNA.

### 2.8. Transforming Growth Factor-β (TGF-β) Production

**Figure 8:**
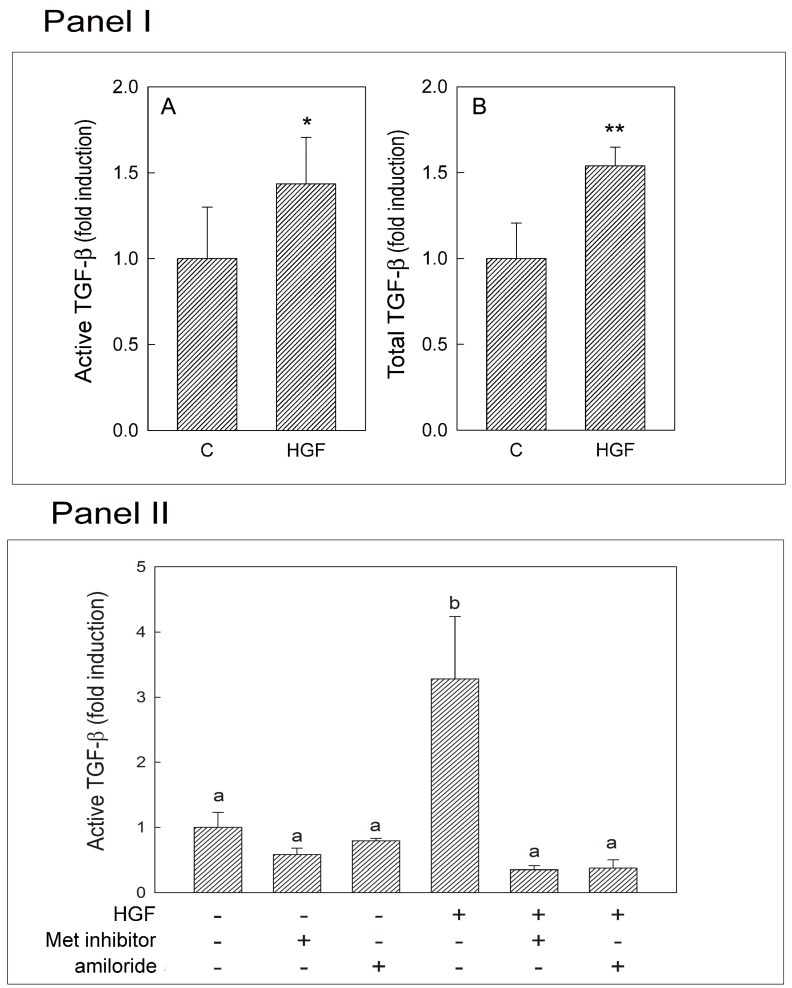
HGF dependent TGF-β secretion and activation in purified myoid cells and in cultured seminiferous tubules. (**I**): TGF-β secreted by purified myoid cells. The amounts of active (**A**) and total (**B**) TGF-β in the control C and HGF-treated samples are shown. The values represent the mean ± SEM of four different experiments and are expressed as fold induction with respect to the control, which was arbitrarily set to one. * *p* < 0.05; ** *p* < 0.01; (**Panel II**): Active TGF-β secreted by cultured tubular fragments at Stages VI–VIII of the seminiferous epithelium cycle. The amounts of active TGF-β secreted in the control condition and after HGF administration are shown. The effect on TGF-β activation due to amiloride or Met inhibitor administered alone or in combination with HGF is also reported. The values represent the mean ± SEM and are expressed as fold induction with respect to the control, which was arbitrarily set to one. a *vs.* b *p* < 0.01; the same letters indicate values that are not statistically significant.

Myoid cells were cultured for 24 h in control medium or in medium supplemented with 150 U/mL (45 ng/mL) HGF, and the secretion of total and active TGF-β was evaluated using a bioassay based on mink lung epithelial cells (MLECs), as described in the Methods Section. The results obtained indicated that HGF significantly increased the secretion of both total and active TGF-β (Figure 8, Panel I). The same assay was performed on tubular fragments isolated at Stages VI–VIII and cultured in the control condition, in the presence of uPA or Met inhibitor alone or in combination with HGF. Actually, we already reported that active TGF-β increases after HGF administration to tubular fragments [11]. Herein, we confirm this result, adding new information. In fact the co-administration of HGF with Met or uPA inhibitors abolished the HGF-dependent TGF-β activation. Furthermore, Met inhibitor alone was able to reduce the mean level of active TGF-β, even though the differences are not statistically significant. This result suggests that the HGF-mediated TGF-β activation is physiological (Figure 8, Panel II).

## 3. Discussion

Rat spermatozoa are shed from the seminiferous tubules at the end of Stage VIII of the seminiferous cycle and are transported toward the rete testis to the epididymis, where they acquire motility. This movement has been postulated to occur by the secretion and flow of fluid in the seminiferous tubules and to be triggered by the contractile activity of the tubules themselves. Myoid cells, which surround the seminiferous tubules, are responsible for tubular contractility, and a variety of substances have been shown to affect their activity, including oxytocin [30], vasopressin [15], prostaglandins [16] and endothelin [17,18]. We previously demonstrated that myoid cells cultured in the presence of HGF modify their morphology, changing from a round, compact shape to an elongated, enlarged shape [8]. In the present work, we investigated the capability of HGF to induce actin cytoskeleton remodeling and to modulate myoid cell contraction. This effect was studied both in cells isolated from prepubertal testes and in peritubular myoid cells of seminiferous tubules from rats of different ages. A change in myoid cell cytoskeletal organization was detected in all samples treated with HGF. In particular, isolated myoid cells showed an HGF-dependent actin cytoskeletal rearrangement highlighted by the formation of actin filament stress-fiber bundles, whereas myoid cells of HGF-treated tubular fragments showed centrally-located actin fibers forming evident rings, which resemble the actin organization of contracted myoid cells [17].

We noted that in tubular fragments from prepubertal rats, HGF clearly induced a myoid cell F-actin reorganization that was fairly homogeneous in the whole peritubular compartment. In contrast, in pubertal and adult tubules, we observed that the arrangement of actin cytoskeleton sharpened in the spotted areas of the peritubular compartment, suggesting a local regulation of responsiveness to HGF in pubertal and adult myoid cells.

To better understand this phenomenon, tubules from adult testes were isolated at the different stages of the seminiferous epithelium cycle. Intriguingly, changes in the actin cytoskeleton were mainly observed in tubules cultured at Stages VI–VIII, and it is worth mentioning that this effect did not occur when Met inhibitor was administered. We hypothesized that a differential response to HGF may reflect differences in Met expression along the tubules. Therefore, we analyzed Met expression at the peritubular myoid level in the different stages of the seminiferous epithelium cycle. We found that the pattern of Met distribution in the myoid cells of prepubertal and adult tubular fragments appeared similar in all of the samples analyzed and was independent of the age and the stage of the seminiferous epithelium cycle, although Met fluorescence intensity was not homogeneous in the neighboring myoid cells. On the basis of these results, it is conceivable to hypothesize that the differential myoid cell responsiveness to HGF might be due to the physiological availability of the factor and/or other cytokines produced that affect the contractility of the myoid cell lineage. In this regard, we have demonstrated that HGF is maximally expressed at Stages VII–VIII of the seminiferous epithelium cycle [11], which is the same stage in which myoid cells appeared to respond to HGF administration. Moreover, the reported HGF mediated-myoid cell contraction might also be mediated by the HGF-dependent upregulation of other factors known to trigger smooth muscle cell contraction. Therefore, the control of differential tubular contractility might be obtained by the regulation of the secretion of the factors involved in this process. Because we previously reported that the treatment of isolated tubules with HGF induced an increased secretion of uPA together with an increase in TGF-β activation [11], we decided to study the uPA dependence of HGF-mediated myoid cell contraction.

Like other proteins, both PAs have been shown to be secreted in a cyclic fashion during the cycle of the seminiferous epithelium [31,32], and this control appears to occur at the mRNA level. High levels of uPA mRNA were found at Stages VII–VIII, whereas the highest levels of tPA mRNA were identified at Stages IX–I [33,34].

The production of uPA at Stages VII–VIII has been suggested to be involved in the extensive tissue remodeling that takes place during the release of the preleptotene spermatocytes from the basement membrane [35], spermiation [36] and the detachment of residual bodies from the mature spermatids [37].

uPA and the interaction with its receptor, uPAR, have been positively implicated in reorganization of the actin cytoskeleton and the adhesion of vascular smooth muscle cells [29]. Moreover, uPA can activate inactive plasminogen, forming the broad-spectrum serine protease plasmin, which, in turn, facilitates the conversion of latent TGF-β to the active form; this is based on the fact that plasmin has been reported to catalyze this conversion [23]. In addition, it has been demonstrated that receptor-bound uPA is required for the plasmin-dependent cellular conversion of latent TGF-β to active TGF-β [38]. TGF-β has been demonstrated to stimulate the assembly of F-actin microfilaments to form bundles of stress fibers on peritubular cells in culture and also induces an increased contractility of peritubular cells embedded in collagen gels [39].

Consistent with these literature data, herein, we show that HGF is able to mediate an increase of uPA and its receptor, uPAR, in purified myoid cells. Moreover, in the same cultural conditions, we also observed an increased activation of TGF-β after HGF stimulation. Therefore, the reported secretion of uPA by myoid cells, as well as the presence of its receptor, uPAR, might be relevant to the process of TGF-β activation. Interestingly we previously demonstrated that HGF was able to increase the uPA level also when administered to adult tubular fragments at the Stages VII–VIII of the seminiferous epithelium cycle [11]. Indeed, when PA activity was inhibited by aprotinin or amiloride, we abolished the contractile effect of HGF, indicating that this effect was mediated by this enzyme. Moreover the use of amiloride, as well as the Met selective inhibitor abolished the HGF-dependent TGF-β activation. In light of this result, we indicate that the observed HGF-mediated uPA activity increase influences the active TGF-β secretion and might thus be responsible, at least in part, for the increased contractility of HGF-treated myoid cells. Further investigation is needed to clarify the physiological pathway crosstalk among HGF and the other cytokines known to regulate myoid cell contraction.

In conclusion, in this study, we provide evidence that: (1) Met is expressed by myoid cells of prepubertal, pubertal and adult rat seminiferous tubules; (2) HGF activates testicular myoid cell cytoskeleton remodeling and contraction; (3) in the adult, myoid cell contraction, due to HGF administration, is sharp only in tubules cultured at Stages VI–VIII of the seminiferous epithelium cycle, which are the stages when myoid cell contraction physiologically occurs, and Met inhibitor administration abolishes this phenomenon; (4) also aprotinin and amiloride, which are uPA inhibitors, abrogate HGF-induced myoid cell contraction; (5) HGF is able to increase the expression level of *uPA* and *uPAR* in purified testicular myoid cells; (6) Met inhibitor administration on tubular fragments abolish the HGF-dependent uPA increase; (7) HGF is able to increase the secretion of active TGF-β, which, interestingly, is one of the factors known to be involved in peritubular cell contraction and can be activated by uPA; and (8) Met or uPA inhibitor administration abolishes HGF-dependent TGF-β activation.

On the basis of these results, we propose to include HGF in the number of factors able to locally modulate, directly and/or indirectly via TGF-β activation, testicular myoid cell contraction.

## 4. Materials and Methods

### 4.1. Animals

Wistar rats were housed under controlled temperature (25 °C) and light (12 h light/day) conditions with *ad libitum* access to food and water at the Sapienza University of Rome, Rome, Italy. All animal studies were conducted in accordance with the Italian Department of Health Guide for Care and Use of Laboratory Animals. The protocol (ID 149321853903) was approved by the Committee on the Ethics of Animal Experiments of the “Sapienza” University of Rome 19 November 2013 (legal provision art. 116/92). Rats were sacrificed by CO_2_ asphyxia before testis removal. Typically, nine or ten 17–18-day-old rats and three or four 28–60-day-old rats were used for each experiment.

### 4.2. Cell Preparation and Culture

Purified myoid cells were prepared according to the method of Palombi *et al.* [40]. In brief, small explants of decapsulated testes isolated from 17–18-day-old rats were digested for 30 min at 32 °C by 0.25% trypsin in PBS to detach the interstitium. The seminiferous tubules were sedimented by gravity, and the supernatant was removed and centrifuged to sediment the interstitial cells. The seminiferous tubules were treated with collagenase A (1 mg/mL) for 30 min at 32 °C to detach the total peritubular cells and then again sedimented by gravity. The supernatant was removed, and the tubules were washed with PBS and sedimented again. The supernatant was removed, pooled with the first and centrifuged for 2 min at 40× *g*. The pellet was further digested in 0.1% trypsin in PBS supplemented with 2% EDTA to obtain a single cell suspension, which was applied to a discontinuous Percoll gradient and centrifuged for 20 min at 800× *g* at room temperature. The fraction corresponding to the myoid cells (density 1.075 g/mL) was collected, and the cells were washed twice with MEM. The purity of the cells used for the experiments was assayed by the presence of alkaline phosphatase activity [40] and was never lower than 94%. For assessment of the effects of HGF *in vitro*, cells were cultured in plastic dishes for 24 h at 32 °C in a humidified 5% CO_2_/95% air atmosphere in the presence of HGF (150 U/mL= 45 ng/mL). At the end of the culture, the conditioned media were collected and used for the PA and TGF-β assays. Myoid cells were either fixed in 4% paraformaldehyde (PFA) and used for F-actin detection or solubilized in lysis buffer for RNA extraction (see below).

### 4.3. Isolation of Seminiferous Tubule Fragments

Tubules were prepared as we previously described, with minor modifications to better preserve the peritubular myoid layer [11,20]. The albuginea of the testes of 17/18- and 35-day-old rats were dissected, and the seminiferous tubules were mechanically isolated and digested with 0.25% and 0.2% trypsin, respectively, in Hank’s buffer without Ca^2+^ and Mg^2+^ at 32 °C in a shaking water bath (90 cycles/min). After 3–5 min, the enzyme was diluted with Hank’s buffer, and the seminiferous tubules were collected by sedimentation (5 min). The albuginea of the testes of 60-day-old rats were removed, and the seminiferous tubules were mechanically dissected without enzymatic digestion. Tubules at Stages VII–VIII of the seminiferous epithelial cycle were isolated according to the method of Parvinen and Vanha-Perttula [41]. Tubules of all ages considered were washed three times with Hank’s buffer and three times with MEM to eliminate the interstitial tissue. To determine myoid cell layer integrity, some of the tubules were assayed by the presence of alkaline phosphatase activity [40]. The isolated tubules were cultured for 24 h at 32 °C in a humidified atmosphere of 5% CO2 in air. These samples were cultured in control medium and in medium supplemented, when indicated, with 150 U/mL (45 ng/mL) HGF, 15 µM aprotinin, 100 µM amiloride and 30 and 60 nM PF-04217903 (Sigma–Aldrich Corp., St. Louis, MO, USA), which is a Met-selective inhibitor. These compounds, when indicated, were added in combination. The concentration of amiloride was used according to Zhu *et al.* [42]. The working concentration of the Met inhibitor was determined by a dose response scatter assay carried out on MDCK cells [8]. In this assay, we used PF-04217903 at concentration of 5, 10, 20, 30, 60 nM and 1 µM. We found that 30 nM is the lowest concentration of PF-04217903 able to inhibit HGF-induced MDCK scatter activity. After culture, the media were collected and used for TGF-β bioassay and PA zymography (see below), whereas the tubules were washed twice in PBS and fixed in 4% PFA for 2 h at 4 °C for immunofluorescence experiments and confocal microscopy analyses.

### 4.4. Met Immunofluorescence

For Met detection, fixed tubules were rinsed three times in PBS. To determine myoid cell layer integrity, some of the tubules were assayed by the presence of alkaline phosphatase activity [40]. Non-specific antibody binding was blocked by rinsing samples for 1 h in PBS with 3% BSA and 0.1% Triton X-100. The seminiferous tubules were then incubated overnight with anti-Met antibody (sc 162G m-Met SP260, 1:1 dilution, Santa Cruz Biotechnology, Heidelberg, Germany). Samples were then washed three times in PBS with 1% BSA and 0.1% Triton X-100. The secondary antibody (FITC-conjugated donkey anti-goat IgG; 1:200 dilution) was incubated for 1 h at RT. Negative controls were treated with secondary antibody only. After secondary antibody incubation, tubules were washed three times in PBS, mounted in buffered glycerol (pH 9) and visualized using the confocal microscope.

### 4.5. F-Actin Detection

For F-actin visualization, fixed purified myoid cells and seminiferous tubules were rinsed three times in PBS for a total of 15 min. Samples were then permeabilized with 0.1% Triton X-100 in PBS for 2 h, rinsed three times in PBS and treated with rhodamine-phalloidin (R415) (Life Technologies, Molecular Probes, Eugene, OR, USA) at 25 μL/mL for 20 min. After incubation with phalloidin, samples were washed three times in PBS and mounted in buffered glycerol (pH 9). Seminiferous tubules were visualized using the confocal microscope, whereas myoid cells were photographed with a Zeiss Axioscope microscope.

### 4.6. Confocal Microscopy

For actin and Met detection, a Leica confocal microscope (Laser Scanning TCS SP2, Leica, Wetzlar, Germany) equipped with Ar/ArKr and He/Ne lasers was utilized. The images were acquired using the Leica confocal software. The laser line was at 488 nm for FITC excitation (Met) and 543 nm for rhodamine excitation (actin). The images were scanned under a 20× objective. Isolated seminiferous tubules were analyzed using optical spatial series with a step size of 1 μm. Images were taken at the peritubular myoid cell layer and at the central part of the tubules.

### 4.7. RNA Extraction and RT

Total RNA from cultured myoid cells was isolated using a silica gel-based membrane spin column (RNeasy Kit, Qiagen S.p.A., Hilden, Germany). The purity and integrity of the RNA was assessed spectroscopically and by gel electrophoresis. Total RNA (1 µg) was reverse transcribed in a final volume of 20 µL using the M-MLV Reverse Transcriptase kit (Invitrogen, Waltham, MA, USA), according to the manufacturer’s instructions.

### 4.8. Multiplex PCR

To determine the presence of the uPA, tPA, uPAR and PAI-1 transcripts, a duplex PCR was performed. The reactions were performed using a Multiplex PCR Kit (Qiagen, Hilden, Germany) according to the manufacturer’s instructions, with the housekeeping gene, GAPDH, used as an internal control.

The primer sequences chosen are shown in Table 1. Each primer pair was previously tested alone for specific amplification. For each sample, 10 μL of PCR product was then subjected to electrophoresis on a 2% (w/v) agarose gel and stained with ethidium bromide. The densitometric evaluation of the bands was performed with AIDA software (Advanced Image Data Analyzer 2.11 raytest GmbH, Straubenhartd, Germany). The relative mRNA levels were normalized against the expression of the housekeeping gene. DNA contamination controls were performed using gene-specific primers on RNA without reverse transcriptase treatment.

**Table 1:** Sequence of oligonucleotides used as RT-PCR primers.

Gene	Primers	Product Length	Reaction Conditions	Gene Bank Accession Number
*GAPDH*	Fw 5'-TTCAACGGCACAGTCAAGGCT-3'	552 bp		NM_017008.4
	Rv 5'-ATTGGGGGTAGGAACACGGAA-3'			
*uPA*	Fw 5'-CCCTGCCTGGCCTGGAATTC-3'	664 bp	Ann: 59 °C, 30"	NM_013085.3
	Rv 5'-CCAAACGGAGCATCACCAAACC-3'		Elong: 72 °C, 30"	
*uPAR*	Fw 5'-GAAGACCAACAGGACCATGAGC-3'	462 bp	Ann: 55 °C, 30"	NM_134352.1
	Rv 5'-CATCCAAAGGTGCTGTTCCC-3'		Elong: 72 °C, 30"	
*tPA*	Fw 5'-GGGGGTACGTGTCAGCAGGCC-3'	621 bp	Ann: 62 °C, 30"	NM_013151.2
	Rv 5'-CGCAGGTGGAGCATGGGGAC-3'		Elong: 72 °C, 30"	
*PAI-1*	Fw 5'-CTGGTGCTGGTGAACGCCCTC-3'	338 bp	Ann: 59 °C, 30"	NM_012620.1
	Rv 5'-CAGGATGAGGAGGCGGGGC-3'		Elong: 72 °C, 30"	

Fw, forward primer; Rv, reverse primer.

### 4.9. Bioassay for TGF-β

Active TGF-β was assayed according to the method of Abe *et al.* [43], which utilizes a mink lung epithelial cell (MLEC) line stably transfected with the luciferase gene. Conditioned media were prepared from myoid cells and tubular fragments cultured for 24 h as described above. Conditioned medium (100 μL) was then added to subconfluent MLECs. After 16 h of culture at 37 °C in 5% CO_2_, MLECs were washed twice with phosphate-buffered saline (PBS), lysed in 60 μL of lysis buffer (Promega, Madison, WI, USA) and stored at −80 °C until use. Cell extracts were assayed for luciferase activity using a kit obtained from Promega and a Netzschalter 090003 luminometer (GSG Nuclear, Milan, Italy). To measure total TGF-β, latent TGF-β was activated by treating the conditioned medium at 80 °C for 6 min, and the media were assayed for TGF-β activity. Parallel MLECs were incubated with known increasing concentrations of TGF-β (Sigma T1654, ranging from 0.0625 to 4.0 ng/mL) to obtain a standard curve.

### 4.10. Gel Electrophoresis and Zymography

For zymography of the plasminogen activator (PA), 10 μL of conditioned medium were separated by 10% sodium dodecyl sulfate polyacrylamide gel electrophoresis (SDS-PAGE) under non-reducing conditions according to the procedure of Laemmli [44]. Molecular weights were calculated from the position of prestained molecular weight markers (Bio–Rad, Milan, Italy, 161-0373) subjected to electrophoresis in parallel lanes. PA was then visualized by placing the Triton X-100 washed gel on a casein-agar-plasminogen underlay, as previously described [25,45]. All of the bands were plasminogen dependent. The densitometric analysis of the band was performed to obtain semiquantitative estimation of protease activities
